# 

**Published:** 1887-03-19

**Authors:** 


### The Hospital.

**An Institution, Family, and Parochial Journal of Hospitals, Asylums, and all Agencies for the Care of the Sick, Criticism and News.**

SATURDAY, MARCH 19TH, 1887.

### THE QUILTER PRIZES.

### HOSPITAL HOUSEKEEPING AND MANAGEMENT.

We offer two prizes of Ten Guineas and Five Guineas respectively for the two best reports on the Expenditure of any single hospital, such reports to give all the details concerning the cost of food and stimulants, brought out in detail in the Table on page ii of the Special Supplement we published on February 5th, together with a critical examination of the various recommendations contained in the Report of the Special Committee of the Newcastle Infirmary, and the addition of the figures to the following heads of expenditure. Hospital secretaries may procure a printed form of return, containing all the particulars to be filled up by competitors, on application to the Editor.

1. *Domestic Expenses.*—Soap and Soda; Laundry, Repairs, and Washing; Water Rate; Coals, Coke, and Wood; Gas and Oil; Bedding and Linen; Furniture, Earthenware, and Hardware; Uniform and Livery, for (*a*) Porters, (*b*) Sisters and Nurses.

2. *Surgery and Dispensary.*—Drugs; Surgical Dressings and Bandages; Instruments, Artificial Limbs, and Trusses; Mineral Waters; Dispensary Sundries.

3. *Salaries and Wages.*—(*a*) Management, including Secretary, Superintendent, Clerks, and Collectors (including commission); (*b*) Nursing Department, Matron or Lady Superintendent, Superintendents of Nursing, Sisters, Nurses, Extra Nurses, Ward Maids and Scourers; (*c*) Maintenance of Porters and Servants (not included in *a* and *b*).

4. *Management Expenses.*—Printing and Stationery; Advertisements; Postage; Special Appeal for Funds; Annual Court and Festival Dinner; Auditor's Fee; Banker's Interest.

5. *Rates and Insurance* (exclusive of Water and Gas rates).

6. *Pensions*, showing to which Department they belong.

7. *Repairs and alterations of Structure.*—(*a*) General, including flooring and painting; (*b*) Extraordinary, including building.

8. *Miscellaneous Expenses* (*i.e.*, all other items to be given in detail).

All the figures are to be those for the year 1886, and the totals must agree with the figures published in the annual report, and copy of which must be sent in with the manuscript. Competitors must enclose their name and address, must write on one side of the paper only, and give the name of the hospital they have selected. No competition should occupy more space than that represented by three pages of printed matter in The Hospital.

All competitions must be sent in not later than the 30th of April next, addressed to the Editor, The Hospital, Norfolk House, Norfolk Street, London, W.C., and it is hoped that particulars may in this way be supplied from every British Hospital.

---

### A GOOD EXCHANGE.

A refuge where those wearied out with pain,
Are taken in and cared for tenderly,
Are helped to win their lost health back again,
By gentle hands that treat them skilfully;
Where kindly ministers and words of cheer,
Give them fresh hope, for dark despair and fear.

Mater.

### THE LITERARY PRIZE.

A PRIZE of **Two Guineas** will be given every month for the best story or incident based upon hospital, or asylum, or student life, or any incident connected with the professions of medicine or nursing.

### The Children's Corner.

### OUR PASTIMES.

### Twelfth Competition.

No. 62. CONUNDRUM. I went to the wood and got it; I sat down to look for it, and brought it home because I could not find it.

No. 63. TRANSPOSITION PUZZLE. The following letters set n right order will reveal the names of three of Shakespeare's heroines, Jetuil; Solinard; Naimard.

No. 64. REBUS. Who can read the following sentence: "The B B him flew and stung him, stung him, stung him."

No. 65. BURIED NAMES. I am two words, of eleven letters in all. My 1, 10, 11, 3 is a story; my 6, 5, 10, 7 is what we use in washing; my 7, 8, 9 is a deep place; my 9, 4, 3 is an article; my 2, 10, 9 is something we wear.

No. 66. ENIGMA.

I saw a peacock with a fiery tail;
I saw a comet shower down hail;
I saw the clouds with ivy bound;
I saw an oak creep upon the ground;
I saw an ant swallow up a whale;
I saw the sea brim full of ale;
I saw a drinking glass full twenty fathoms deep;
I saw a well full of tears that men do weep;
I saw men's eyes at twelve o'clock at night;
I saw the man who saw this wondrous sight.

(How can the above be made possible without altering the order of any of the words?)

### Results of Tenth Competition.

No. 52. A PUZZLE IN SUBTRACTION. If you take I from XIX, it leaves XX (twenty).

No. 53. CONUNDRUM. Why was Noah the greatest financier of his time? Because, you know, he kept a *limited company* afloat, when all the rest of the world was in *liquidation*.

No. 54. FLOWERS ENIGMATICALLY EXPRESSED. These were the Fox-glove, Blue-bell, and Butter-cup.

No. 55 ARITHMETICAL PUZZLE. 100+1+5+1+50 seems to have easily metamorphosed into CIVIL.

No. 56. HIDDEN CHRISTIAN NAMES. Nina, Ted, Rose, Tom, Sal, Dan, Ned, Ada, Ernest, Amy, Eve, Arthur, Hal, (Lisa, Alma, Eric, Rica, Isa, Thea, and one or two other rare names may be found in this puzzle).

*Five right*—A. C. K., Alsie, Scrooge, P.S., Florrie, Skye, Peeler, Mikado, Paul Methven; *Four right*—Dot, Agatha, Tim, Ellie, Joe; *Three right*—Isobel, Little Mary, Bismarck, Ariadne.

### Letters from Little Folk.

The subject for next week will be "The Town in which I Live." Tell me anything about it that is instructive or interesting, such as its old buildings, churches, pretty walks in the neighbourhood, or anything else you think of. The letters about "Amusements" display varied tastes Peeler extols the virtues of cricket, which he truly styles the "favourite game of every Englishman." Here is a little epistle from Halford on his favourite amusement, a most useful one, I must confess:

DEAR MR. EDITOR,—My chief amusement is carpentering. I have the carpenter in every Saturday afternoon from four till five o'clock, which I enjoy very much. Another amusement of mine is fishing; we caught five perch in one day.

Yours truly, HALFORD, aged 9.

The Lodge, Porchester Square, W.

Besides cricket, Tim goes in for football and paper chases. Two little girls acknowledge their partiality for cricket, although, adds one of them (Florrie), "it is a boy's game." Here is what Isobel says anent this game and other matters:

DEAR MR. EDITOR,—I think the amusements I like best are cooking and cricket, "I spy," and spelling games. I have a beautiful gas stove that I cook on in our schoolroom. It will cook puddings, omelettes, vegetables, chops, beefsteaks, and toast. Cricket I play with my brother; we are both fond of it, and I think "I Spy" is great fun out of doors where there are plenty of good places to hide in. We play a good many spelling, letter, and verse-making games, and also chess and draughts, in the winter evenings.

Yours truly, ISOBEL, aged 15.

Vicars' Court, Lincoln.

Mikado also favours the culinary art, and makes beautiful cakes. Dancing is also another favourite amusement of hers. Scrooge likes "Hide and Seek," and "Rounders." Tennis she also took up last summer and so too did Florrie. Skye's tastes, I know, lie in the direction of reading and drawing, but I had no idea until I received the following revelations, that donkey rides were so much in the ascendant.

DEAR MR. EDITOR,—My favourite amusements are drawing and donkey riding. There is nothing I like so much as a gallop on a donkey at the seaside. Last year when we went, I spent every bit of pocket-money I had on donkey rides. Whenever we get to the seashore, the first thing I do is to look about for a donkey. I used to like digging in the sand, but I do not now. Then I like drawing very much when I have time. I have not very much time now, as I am going in for the Cambridge examination next year, and I have a good many lessons to do. I like working in the garden very much. I sowed a great many flower-seeds last year, and they all came up very well. Another great amusement is finding out your puzzles.—Yours faithfully, SKYE, aged 14.

4, Milton Terrace, New Road, Chatham.

### Letter Prize.

One mark each: Tim, Peeler, Florrie, Mikado, Scrooge, Halford, Isobel, Ellie, Skye.

I am glad to see from my weekly budget, that my puzzles so generally take rank among the amusements of my dear little friends.

---

Answers must be addressed to the "PUZZLE EDITOR," THE HOSPITAL, Norfolk House, Norfolk Street, Strand, W.C., not later than Thursday next. The "Children's Corner" Coupon (see advertisement pages) must be enclosed.

The address and full Christian name and surname, and the age of the competitor, must be stated in all replies. A *nom de plume* may be added for the purposes of publication.

### Amusements with Profit.

### PRIZE COMPETITIONS.

### Quarterly Prize Competition.

Began 22nd January; Ends 16th April.

### A Prize of Three Guineas will be awarded to the Competitor who shall have sent in the largest number of correct or best answers. No one will be permitted to win the Three Guinea Prize twice in any one year, but we shall award annually an additional

### Prize of Five Guineas to the competitor who shall have sent in the largest number of correct or best answers during the twelve months.

### No. 17. Double Acrostic.

These houses of mercy for the suffering young
Have in quite recent years into existence sprung.

A famous Greek the *art* of *Æsculapius* taught;
A man who the life of the third *Napoleon* sought;
A *law* which lost to us a land we held till then;
A novelist's *creation*, and a well-known *pen*;
A word pertaining to types similar to *these*;
An *ornament* found in the Doric column frieze;
A class of hermits strange, who swathed themselves in *white*;
A kind of Dixie's land in ev'ry *Scotchman's* sight;
A prince's son, 'tis Cambria's *patron saint* you'll find;
An *ascetic monk* in the distant realms of Ind;
A branch of this craves peace—why, *Noah's story* saith;
A *bird* of omen bad, which bodes ill-luck or death.

### No. 18. "Hospital."

Give, in prose, a brief account of the word "hospital," its origin and the ultimate application of the term to its present use. Credit will be given for those answers which trace the history of the word with succinctness and accuracy. They may be illustrated by quotations.

### Answers.

No. 13. DOUBLE ACROSTIC.

S ina I

H ibernia N

E l F

F ast I

F orfa R

I mau M

E tn A

L uthe R

D 'Arbla Y

Correct answers have been received from Ostrich, K. M., Peeler, Condorcet, Mr. J. High, Gip, Miss Larkins, Ellice. ("His" was a misprint for "her" in the last "light," but most of our correspondents have correctly given Madame D'Arblay. We have, however, credited all answers where the other lights were correctly given.) We cannot accept "Islam" for the fifth light, since it is the name of the religion, and not the title of a chief (Imaum=teacher).

No. 14. CHARADE.

The town is Oxford.

Correct answers have been received from Miss Larkins, Paddy, Mrs. Bedingfeld, Gip, Condorcet, Peeler, K. M., Ellice, Ostrich, Mr. J. High, Frederick C., Tabs, Gretta, Patient, Mater, Robe, Perseverance, A. M. E.

### Notices to Correspondents.

OSTRICH.—We have no objection whatever.

---

Answers must be addressed to THE PRIZE EDITOR, AMUSEMENTS WITH PROFIT, THE HOSPITAL, Norfolk House, Norfolk Street, Strand, W.C., not later than the *first* post on Saturday next. The full name and address must in all cases be given, but a *nom de plume* may be added for publication. Only one side of the paper should be written on. The "Amusements with Profit" Coupon (see advertisement pages) must be enclosed with replies.

### The In- and Out-Patients' Hospital Diary.

This Diary is so arranged as to make some portion of it appear every week, and the whole in each monthly part. It has been very difficult to compile, and corrections will at all times be gratefully received. It has been suggested that similar information about the larger Provincial and Scotch Hospitals would be useful to many readers. We should be glad to hear if this is felt to be the case.

### CHILDREN'S DISEASES.—*continued.*

| Hospitals and Terms of Admission. | Physicians, Surgeons, and Medical Officers, with Days and Hours of Attendance. |
|---|---|
| Samaritan Free Hospital for Women and Children, Lower Seymour Street, W.; Branch, 1, Dorset St., W. Sec., Mr. G. Scudamore. In-patients must be between 2 and 12. Out-patients Department (free) is at Edward's Mews, Duke Street, W. | *In-Physician*, Dr. W. H. Day. Daily, 2; *In-Surgeon*, Mr. W. A. Meredith. Daily, 2; *Out-Physicians*, Drs. M. Prickett and Amand Routh, W. S. 1.30; *Out-Surgeons*, Messrs. W. A. Meredith and J. D. Malcolm, M. Th.; A. H. G. Doran and W. S. A. Griffith, Tu. F., 1.30 |
| Victoria Hosp. for Children, Queen's Road, Chelsea, S.W. Sec., Capt. W. C. Blount, R.N. Age of boys, 2 to 12 years; age of girls, 2 to 16. Admission for in-patients by subscriber's letter. Accidents and urgent cases free at all times | *In-Physicians*, Drs. J. Evans, M. Th. 2; Ridge-Jones, Tu. F. 2; *In-Surgeons*, Messrs. Pick, Tu. F. 9.15; Clutton, M. Th. 4.30; *Out-Physicians*, Drs. A. Venn, W. S. 9.30; C. Fox, M. Th. 1.30; F. D. Drewitt, Tu. F. 9; J. Philpot, M. Th. 9; *Out-Surgeons*, Messrs. F. Churchill, Tu. F. 10; W. Pye, M. Th. 9.30 |
| West London Hospital, Hammersmith rd, W. | Daily, 2. *See* General Hospitals |

### CONSUMPTION AND DISEASES OF THE CHEST.

| Hospitals and Terms of Admission. | Physicians, Surgeons, and Medical Officers, with Days and Hours of Attendance. |
|---|---|
| City of London Hospital for Diseases of the Chest, Victoria Park, E. Sec., Mr. T. Storrar Smith. By subscriber's letter available for 6 weeks. | *In-Physicians*, Drs. J. Thorowgood, S.; E. Smith, M.; J. Fothergill, W.; S. West, F.; G. A. Heron, W.; V. D. Harris, Tu. Hour, 2; *Out-Physicians*, Drs. V. D. Harris, Tu.; J. A. Ormerod, Th.; E. C. Beale, Tu. F.; B. Fenwick, W. S.; A. Money, W. S.; L. E. Shaw, M. Th. Hour, 2 |
| Hospital for Consumption, Fulham road, Brompton, S.W. Sec., Mr. H. Dobbin. Admission by letter of recommendation, but, where unobtainable, application may be made to the Secretary at the Hospital. | *In-Physicians*, Drs. E. S. Thompson, M. Th.; C. T. Williams, M. Th.; R. D. Powell, Tu. F.; J. Tatham, W. S.; R. E. Thompson, W. S.; F. T. Roberts, Tu. F. Hours, 2 to 4; *In-Surgeon*, Mr. R. J. Godlee, F.; *Out-Physicians*, Drs. T. H. Green, J. M. Bruce, M. Th.; J. K. Fowler, W. S.; P. Kidd and C. Y. Biss, Tu. F.; T. D. Acland, W. S. Hour, 12 |
| North London Hospital for Consumption and Diseases of the Chest, Mount Vernon, Hampstead, N. Sec., Mr. L. F. Hill. Admission by subscriber's letter available for 6 weeks. | *In-Physicians*, Drs. A. Evershed, Tu.; B. O'Connor, F.; D. Pidcock, F. Hour, 10.30; *Out-Physicians*, Drs. B. O'Connor, D. Pidcock, J. E. Squire. Daily, 2 (attend at Out-patients' Branch, 216, Tottenham Court Road, W.) |
| Royal Hospital for Diseases of the Chest, 231, City Rd., E.C. Sec., Mr. John J. Austin. By Governor's letter, available for six weeks | *In-Physicians*, Drs. Hensley, W.; Gilbart-Smith, M.; Finlay, Th. *In-Surgeon*, Mr. A. Pearce-Gould, M. Tu. W. Th. F. 2, S. 9; *Out-Physicians*, Drs. White, Tu. F.; Pringle, M. Th.; O. Browne, W. S.; A. Davies, M. F.; H. Habershon, W. S.; E. Stewart, Tu. Th. Hour, 1; S. 9 |
| Royal National Hosp. for Consumption and Diseases of the Chest, Ventnor. Offices, 34, Craven St., Strand, W.C. Sec., Mr. E. Morgan. Admits only incipient cases by Governor's letter. | *In- and Out-Physicians*, Drs. J. G. Sinclair Coghill, and R. Robertson attend daily (at Ventnor) on out-patients at 11; on in-patients at noon; *In- and Out-Surgeons*, Messrs. Whitehead, Williamson |

### EAR CASES.

| Hospitals and Terms of Admission. | Physicians, Surgeons, and Medical Officers, with Days and Hours of Attendance. |
|---|---|
| Central London Throat and Ear Hosp., Gray's Inn Rd., W.C. Sec., Mr. R. Kershaw. Free, but when able, a small payment is expected | *In- and Out-Surgeons*, Mr. Lennox Browne, M, Th. 2.30; Dr. Orwin, W. S. 2.30; Dr. Grant, Tu. 5, Th. 2.30; Messrs. P. S. Jakins, M. W. 2.30; F. 5; T. Carmalt Jones, M. Th. S. 2.30 |
| Charing Cross Hospital, West Strand, W.C. | *In- and Out-Surgeon*, Mr. Cantlie, F. 9 |
| Great Northern Central Hospital, Caledonian Rd, N. | *In- and Out-Surgeon*, Mr. A. E. Cumberbatch, W. 9 |
| Guy's Hospital, St. Thomas's Street, S.E. | *In- and Out-Surgeon*, Mr. Purves, Tu. F. 12.30 |
| Hospital for Diseases of the Throat, Golden Square, W. Sec., Mr. W. T. Sharp | *Physicians*, Drs. Wolfenden, Tu. F. 2.30; Macdonald, Tu. F. 6.30; Bond, W. S. 2.30. *Surgeon*, Mr. T. M. Hovell, M. Th. 2.30 |

### EAR CASES—*Continued.*

| Hospitals and Terms of Admission. | Physicians, Surgeons, and Medical Officers, with Days and Hours of Attendance. |
|---|---|
| King's College Hospital, Portugal Street, W.C. | *In- and Out- Surgeon*, Mr. Pritchard Th. 2.30 |
| London Hospital, Whitechapel Road, E. | *In- and Out-Surgeons*, Dr. Woakes, Mr. T. M. Hovell, S. 9 |
| Middlesex Hospital, Berners Street, W. Free. | *Out-Surgeon*, Mr. Hensman, Tu. 9 |
| National Hosp. for the Paralysed, etc., Queen Sq., W.C. | *In- and Out-Surgeon*, Mr. A. E. Cumberbatch, W. 2 |
| North West London Hospital, 18, Kentish Town Rd. | *Out-Surgeon*, Mr. Collier, M. 1.30 |
| St. Bartholomew's Hospital, Smithfield, E.C. | *Surgeon*, Mr. Cumberbatch, Tu. F. 2 |
| St. George's Hospital, Hyde Park Corner, S.W. | *In- and Out-Surgeon*, Sir W. B. Dalby, Tu. 1.30 |
| St. Mary's Hospital, Cambridge Place, Paddington, W. | *In- and Out-Surgeon*, Mr. G. P. Field, W. S. 9.30 |
| St. Thomas's Hospital, Westminster Bridge Road, S.E. | *Out-Surgeon*, Mr. Clutton, M. 1.30 |
| University College Hospital, Gower Street, W.C. | *In- and Out-Surgeon*, Mr. Barker, Th. 9 |
| Westminster Hospital, Broad Sanctuary, S.W. | *In- and Out-Surgeon*, Mr. Barrow, Tu. F. 9 |

### EYE DISEASES.

| Hospitals and Terms of Admission. | Physicians, Surgeons, and Medical Officers, with Days and Hours of Attendance. |
|---|---|
| Central London Ophthalmic Hosp., 238A, Gray's Inn Rd., W.C. Sec., Mr. G. H. Leah. Admission free | *Surgeons*, Messrs. T. Archer, G. Abbott, W. Charnley, W. Jessop, E. Clarke, J. R. Kemp and Granville Barker (House Surgeon). Daily, 1 |
| Evelina Hosp. for Sick Children, Southwark Bridge Rd. | *Surgeon*, Dr. W. Brailey, Th. 1 |
| Great Northern Central Hospital, Caledonian Rd., N. | *Surgeon*, Mr. Hutchinson, Jnr., Tu. F. 9.30 |
| Guy's Hospital, St. Thomas's Street, S.E. | *Surgeons*, Mr. Higgens, Dr. Brailey, Tu. F. 12.30 |
| Hospital for Sick Children, 49, Gt. Ormond Street, W.C. | *Surgeon*, Mr. R. Marcus Gunn, Tu. 2 |
| King's College Hospital, Portugal Street, W.C. | *Surgeon*, Mr. McHardy, M. W. Th. 1.30 |
| London Hospital, Whitechapel Road, E. | *Surgeons*, Messrs. Waren Tay, S. 9; Eve, Tu. 9 |
| Middlesex Hospital, Berners Street, W. | *Surgeon*, Mr. Lang, Tu. F. 9 |
| National Hosp. for the Paralysed, etc. Queen Sq., W.C. | *Surgeons*, Messrs. R. Brudenell Carter, F. 4; R. Marcus Gunn, Tu. 4 |
| North-West London Hosp. 18, Kentish Town Rd., N.W. | *Surgeon*, Dr. W. Collins, F. 3.30 |
| Paddington Green Children's Hosp., Paddington Grn. | *Surgeon*, Mr. W. H. H. Jessop, Th. 9 |
| Royal Free Hospital, Gray's Inn Road, W.C. | *Surgeon*, Mr. Mackinlay, M. F. 9 |
| Royal London Ophthalmic Hospital, Blomfield Street, Moorfields, E.C. Sec., Mr. R. J. Newstead. Admission free to the poor. | *Surgeons*, Messrs. J. W. Hulke, W. S.; G. Lawson, Tu. F.; J. Couper, Tu. F.; W. Tay, M. Th.; J. Tweedy, Tu. F.; E. Nettleship, W. S.; R. M. Gunn, W. S.; Lang, M. Th. Hours, 8 to 10 |
| Royal South London Ophthalmic Hosp., 6, St. George's Circus, S.E. Sec., Mr. C. Comyn. Free to the necessitous poor; or by payment | *Surgeons*, Messrs. Watson, McHardy. Daily, 2 |
| Royal Westminster Ophthalmic Hospital, King William Street, W.C. Sec., Mr. T. Beattie-Campbell. Poor and accidents free | *In- and Out-Surgeons*, Messrs. Power, M. F.; Frost, M. Th.; Rouse, Tu. S.; Hartridge, Tu. F.; Macnamara, M. Th.; Cowell, W. S.; Juler, W. S. Hour, 1 |
| St. Bartholomew's Hospital, Smithfield, E.C. | *Surgeons*, Messrs. Power, Th. 2; Vernon, Tu. S. 2.30 |
| St. George's Hospital, Hyde Park Corner, S.W. | *Surgeons*, Messrs. B. Carter, Frost, M. Th. 2; F. 1.15; W. S. 2 |
| St. Mary's Hospital, Cambridge Place, Paddington. W. | *Surgeons*, Messrs. A. Critchett, H. Juler, Tu. F. 9.30 |
| St. Thomas's Hospital, Westminster Bridge Road, S.E. | *Surgeons*, Messrs. Nettleship, Tu. Th. F. 1.30; Lawford, M. W. 1.30 |
| University College Hospital, Gower Street, W.C. | *Surgeon*, Mr. Tweedy, M. Tu. Th. F. 2 |
| West London Hospital, Hammersmith Road, W. | *Surgeons*, Messrs. B. Vernon, H. Dunn, Tu. Th. S. 2 |
| Westminster Hospital, Broad Sanctuary, S.W. | *Surgeon*, Mr. Cowell, M. Th. 2 |
| Western Ophthalmic Hosp. 153 and 155, Marylebone Rd., W. Sec., Mr. G. E. Manton. By letter or small payments; free to poor | *Surgeons*, Drs. Miller, M.; Collins, F.; Wheatly, M. Th.; Messrs. Carmal, Jones, W.; Buxton, Tu. F.; Nunn W. S. Hour, 2 |

